# In Vivo Release Kinetics and Antibacterial Activity of Novel Polyphenols-Enriched Chewing Gums

**DOI:** 10.3390/molecules21081008

**Published:** 2016-08-02

**Authors:** Gianmaria Fabrizio Ferrazzano, Tiziana Cantile, Marco Coda, Brunella Alcidi, Giancarla Sangianantoni, Aniello Ingenito, Michele Di Stasio, Maria Grazia Volpe

**Affiliations:** 1 Department of Neuroscience, Reproductive and Oral Sciences, Section of Paediatric Dentistry, University of Naples, Federico II, Naples 80131, Italy; tizianacantile@yahoo.it (T.C.); marcocoda.od@gmail.com (M.C.); brunella.alcidi@gmail.com (B.A.); giancarla.sangia@yahoo.com (G.S.); ingenito@unina.it (A.I.); 2 Division of Dentistry and Orthodontics, Bambino Gesù Hospital, Rome 00165, Italy; 3 Nutrition Science Institute, National Council of Researches, Via Roma 64, Avellino 83100, Italy; michele.distasio@isa.cnr.it (M.D.S.); mgvolpe@isa.cnr.it (M.G.V.)

**Keywords:** caries, caries prevention, polyphenols, quercetin, chewing gums, release kinetics

## Abstract

Chewing gums may be particularly effective means for delivering and maintaining bioactive molecules, included in the gum formulation, able to have an anti-cariogenic effect. The purposes of this study were: to develop novel chewing gums containing quercetin (Qt); to evaluate their release using in vivo trial; finally, to test their in vivo antibacterial effect against oral *Streptococcus mutans* strains. A preliminary study was performed to produce new gums, enriched with the polyphenol quercetin. Then, a first in vivo experimental study was assessed to test the percentages of Qt released in the saliva of young volunteers. Moreover, a second clinical trial was performed to analyze the antibacterial capability of these enriched chewing gums against *S. mutans* strains after 14 days of daily consumption. The release analysis showed that a more effective release of Qt occurs in the first minutes of chewing, and it does not change saliva pH values. Moreover, Qt included in gums demonstrates an effective antibacterial activity, showing a reduction of the concentration of *S. mutans* strains in saliva samples, especially after 7 days. Qt included in experimental chewing gums could be efficiently released into the oral cavity and could promote an effective anti-caries concentration in volunteer’s saliva, without changing salivary pH values.

## 1. Introduction

Saliva plays multiple roles in oral physiology. Aside from providing a constant rinse, the values of saliva as a reservoir for calcium, phosphate, and fluoride have been well established [1]. The buffering capacity of human saliva plays a major role in countering pH fluctuations. Particularly, bicarbonates in saliva play a great role in elevating low oral pH after meals [2]. Various salivary components also demonstrate antibacterial capability: the iron-binding protein lactoferrin has been shown to inhibit aerobic and facultative anaerobic bacteria (such as *Streptococcus mutans*) which require iron to metabolize. Lysozyme also exhibits direct antibacterial function [3].

In recent years, research is developing new methods of dental disease prevention, releasing natural bioactive compounds included in traditional or innovative medical devices in the mouth to improve saliva protection.

In the pharmaceutical field, using enriched gums, a drug could be released in the mouth from gum and could act either locally or systemically, in which the drug can be absorbed through the buccal mucosa or swallowed with saliva [4]. Using chewing gum could increase the saliva volume and amplify the salivary buffer system, which could neutralize acidic attacks and increase dental biofilm production. Medicated gums can be used for the delivery of dental products in the mouth as an alternative to mouthwashes, or can be used for some diseases such as xerostomia or for other systemic diseases [4]. This drug formulation is intended to be chewed for 10–30 min to allow the ingredient to dissolve in the saliva, prior to swallowing [4]. Some enriched chewing gums introduced in pharmacopeia for their systemic effects are pain killers, vitamins such as vitamin C, alertness enhancers, motion sickness removal, and smoking cessation gums [4].

Because chewing gum is usually kept in the mouth much longer than rinses and toothpastes, the active agent included in a chewing gum formulation—if efficiently released into the saliva—could exhibit sustained and improved delivery in the mouth [5].

In dentistry, aside from new medical devices used to deliver bioactive compounds, nowadays the research is exploring foods naturally rich in bio-molecules and already in use in traditional medicine, considering them as good alternatives to synthetic molecules [6]. Most of the bioactive molecules are derived either directly or indirectly from naturally-occurring sources, especially terrestrial food plants and marine species or from animal products [7,8]. As mentioned above, these natural compounds are being incorporated in pharmaceuticals with the purpose of delivering specific oral health benefits.

In the literature, many plant- and animal-derived products have been analyzed for their effectiveness in the prevention of dental plaque formation. However, only a very small number of these natural products have been used for therapeutic applications, due to various factors such as adequate effectiveness, stability, smell, taste, and cost [9]. The more effective classes of natural biomolecules analyzed for dental disease prevention are polyphenols and milk-derived peptides.

Polyphenols constitute one of the most common and widespread groups of substances in plants, and in medical research it has been shown that several of these may have beneficial effects on humans. The biological properties of polyphenols include antioxidant [10,11], anticancer [12], and anti-inflammatory [13] effects. Experimental studies also strongly support a role of polyphenols in the prevention of cardiovascular disease, osteoporosis, diabetes mellitus, and neurodegenerative diseases [14].

Particularly, polyphenols from some edible plants have attracted attention as potential sources of agents capable of controlling the growth of oral bacteria [15,16,17]; even though the prevalence of dental caries has decreased through the use of conventional preventive systems (fluoride prophylaxis, fluoride toothpastes, control of oral hygiene, sealants) [18,19,20,21], it still remains one of the most common chronic diseases both in health and in systemic disease-affected children [22,23,24,25].

A previous study of the in vitro and in vivo antimicrobial effects of *Plantago lanceolata* herbal tea (from flowers and leaves) on cariogenic bacteria led to the isolation of several polyphenols, including the flavonoid quercetin [17].

Therefore, the objective of this study was to develop a novel, sustained release chewing gum formulation containing quercetin (Qt), a flavonoid derived from plant extraction, well known to have an anti-bacterial activity [25,26,27,28].

The goals of this study were: to produce chewing gums with good technological properties of release; to perform a clinical trial with young volunteers in order to analyze the Qt concentration released in volunteers′ saliva; and to assess its microbiological effect against *Streptococcus mutans* strains in saliva.

## 2. Results

### 2.1. Salivary Flow Rate and pH Analysis

**Figure 1:**
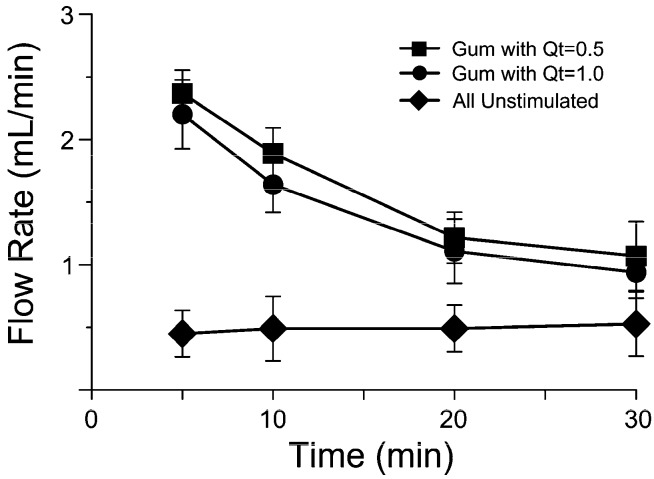
The changes in salivary flow rate (mean ± S.D.) for 32 participants (three samples for each participant, for three experiments) over a 30 min period, collected at 5, 10, 20, and 30 min. Qt: quercetin.

**Figure 2:**
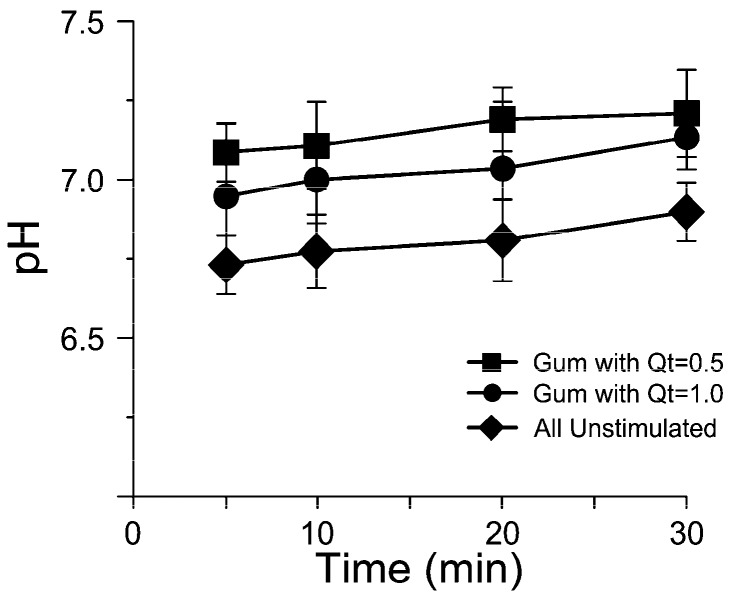
The changes in salivary pH (mean ± S.D.) for 32 participants (three samples for each participant, for three experiments) over a 30 min period, collected at 5, 10, 20, and 30 min.

The results (mean ± S.D.) for flow rate and pH are shown in Figure 1 and Figure 2. During the 30 min session in which saliva was collected, a repeated-measures ANOVA showed that there was no significant change in salivary flow while chewing gums at two concentrations of quercetin; instead, there was a significant difference in comparison with samples of saliva collected during the session without gum chewing.

The mean flow rate of the collection of saliva during the first 5 min of chewing ranged from 2.18 to 2.36 mL/min; it decreased to 1.71–1.84 mL/min and 1.33–1.36 mL/min respectively after 10 min and 20 min of chewing periods, and fell to about 1.04–1.08 mL/min after 30 min, showing a similar trend during the chewing time (Figure 1).

For the session in which only unstimulated saliva samples were collected, the mean pH was relatively constant and in the range of 6.71–6.80. With the chewing gums enriched with 5 and 10 mg of quercetin, the pH increased from a value of 6.84 to 7.11 and from a value of 7.09 to 7.18, respectively (Figure 2); repeated measures analysis of variance (RM-ANOVA) showed that there was no significant change in pH values while chewing gums at two concentration of quercetin, in comparison with samples of saliva collected during the session without gum chewing.

### 2.2. Quercetin Content

The percentages of Qt released into the saliva during mastication were calculated. The assessment of Qt content in saliva was performed with the colorimetric method, as described in materials and methods section.

**Figure 3:**
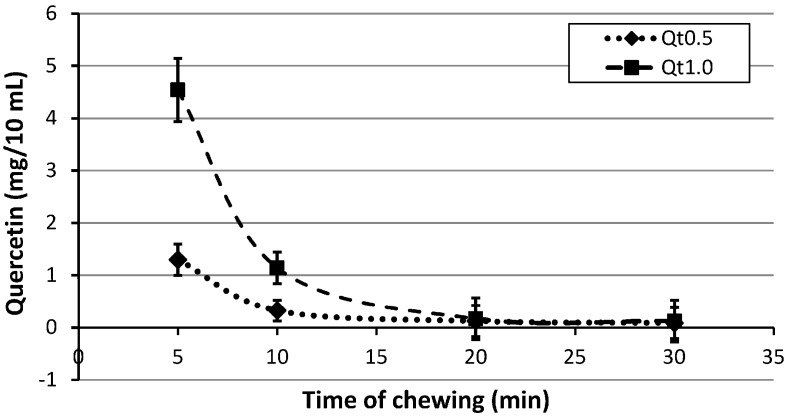
In vivo release of quercetin (mean ± S.D.) from different gum formulations by a chew-out study performed by 32 participants (three samples for each participant, for two experiments) over a 30 min period. Saliva was collected at 5, 10, 20, and 30 min.

The quercetin released, as a function of mastication time from chewing gums containing different amounts (0.5% and 1.0%) of bio-molecule, is reported in Figure 3.

In Qt0.5% samples, the quercetin concentration released was 1.29 mg/10 mL after 5 min of chewing, 0.32 mg/10 mL after 10 min, 0.12 mg/10 mL after 20 min, and 0.09 mg/10 mL after 30 min. Instead, in Qt1.0% samples, collections of saliva showed a Qt release of 4.54 mg/10 mL after the first 5 min, then fell quickly to 1.14 mg/10 mL after 10 min, finally decreasing slowly to 0.17 mg/10 mL and 0.13 mg/10 mL after 20 and 30 min respectively.

### 2.3. In Vivo Assessment of Antimicrobial Activity

**Figure 4:**
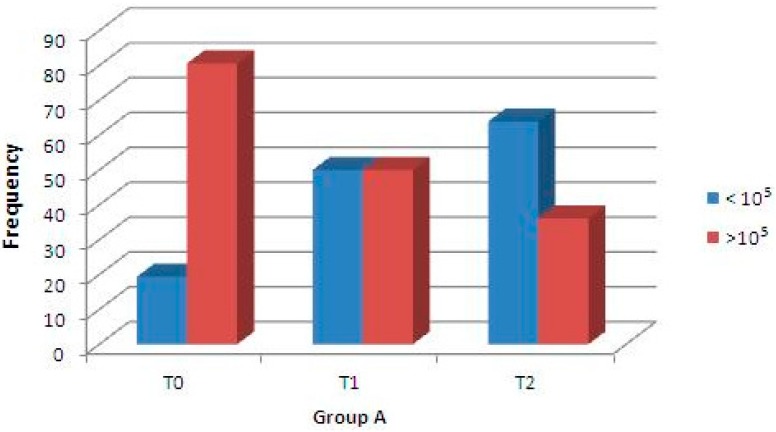
Percentage of Group A samples in relation to density changes of *S. mutans* (CFU/mL) at baseline (T0), after 7 days (T1), and after 14 days (T2). A regression binary logistic analysis showed a significant difference (*p* < 0.001) between T0 and T1, and between T0 and T2.

**Figure 5:**
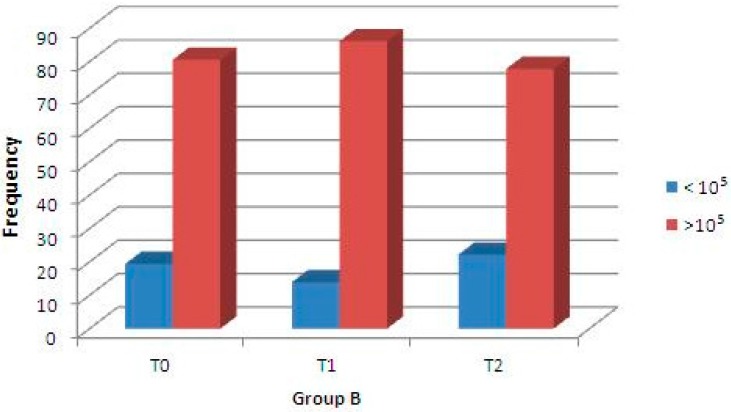
Percentage of Group B samples in relation to density changes of *S. mutans* (CFU/mL) at baseline (T0), after 7 days (T1), and after 14 days (T2). A regression binary logistic analysis did not show a significant difference between T0 and T1, and between T0 and T2.

A second clinical trial was performed to analyze the antibacterial capability of these enriched chewing gums against *S. mutans* strains after 14 days of daily consumption. Concerning Group A samples, using regression binary logistic analysis, the microbiological assay of *S. mutans* concentration (CFU/mL) at baseline (T0) and after 7 days (T1), and between baseline (T0) and after 14 days (T2) showed statistically significant differences (*p* < 0.001 for both, Figure 4); moreover, the difference between *S. mutans* CFU after 7 days (T1) and 14 days (T2) was not significant. Instead, the microbiological assay of Group B (control) showed that differences in the density (CFU/mL) of Streptococci were not statistically significant (Figure 5). Finally, the difference in the streptococci density between Group A (test) and Group B (control) was not significant at T0, but was statistically significant at T1 and T2 (Table 1).

## 3. Discussion

### 3.1. In Vivo Release Kinetics Evaluation

The results of in vivo release kinetics experiments lead us to discuss some aspects. The first consideration is that in adding Qt to gums, there were no differences in the pH of oral saliva: this proves that quercetin mixed with other components of chewing gums could not significantly modify pH values when released in the oral cavity. Therefore, the eventual anticaries capability of experimental gums is not related to their buffering capacity. Moreover, the pH values reported after each time-point always showed not-significant variations in pH values.

Additionally, interesting considerations concerning the release rates for Qt included in chewing gums were observed. In fact, our results are in accordance with several studies showing that chewing gums may be a particularly effective means of delivering and maintaining a sufficient dose of bioactive molecules in the oral cavity [29,30].

In particular, as already shown in vitro by Di Stasio et al. [29], also in the present research the total amount of Qt in vivo released in saliva decreased progressively as a function of the chewing time, regardless of the different concentration of bioactive molecule: it was shown that in 30 min of chewing, the Qt is delivered in a larger amount (70%–90%) and that Qt was quickly released after 10 min of mastication, due to the higher solubility in the artificial saliva—indicating that Qt was released mainly in the first 10 min. Therefore, for this bio-molecule, the more effective release occurs in the first minutes of chewing, and it is compatible with an effective oral action during daily consumption of medicated gums.

### 3.2. In Vivo Antimicrobial Activity of Experimental Gums

In recent years, polyphenols from some edible plants have been identified as potential sources of agents capable of controlling the growth of oral bacteria. In particular, extracts from *Camellia sinensis*, *Helichrysum litoreum*, and *Plantago lanceolata*—rich in flavonoids such as quercetin—have shown inhibitory activity against oral cariogenic Streptococci [31,32,33]. In addition to this aspect, the ability of enriched chewing gums to inhibit *S. mutans* colonies growth is well established in other clinical trials [30,31,32,33,34].

In the present study, the noteworthy innovation is that a natural compound of plants that is safe and biocompatible for humans (i.e., the flavonoid quercetin) included in experimental chewing gums significantly reduces *S. mutans* concentration (CFU/mL): in fact, in the second trial, saliva samples of treated volunteers showed a statistically significant reduction of *S. mutans* CFU after 7 days of gum consumption (Figure 4). This effect is still present after 14 days of treatment, but with a slight reduction. Moreover, this antibacterial effect is not seen in the samples of the control group, and particularly, a statistically significant reduction of *S. mutans* CFU between baseline and after one or two weeks of daily consumption was not present (Figure 5).

In this second trial, the introduction of 10 mg of quercetin in gums was related to its highest release in oral saliva during chewing tests of first trial.

The biochemical mechanism of inhibition of quercetin against microbiological growth is probably based on two different mechanisms of interaction with bacterial DNA or with the ATP binding site of bacterial gyrase [32]. Therefore, it is possible to affirm the middle-time assumption that chewing gums enriched with a phenolic compound could promote an antimicrobial effect against cariogenic bacteria.

These results motivate the need for further studies to establish the long-lasting antimicrobial effect of this treatment and to determine whether resistance will occur. Moreover, the long-term compliance of patients needs to be assessed.

## 4. Materials and Methods

Chewing gum production, biochemical, and microbiological analysis were made by the National Council of Researches (CNR)—Nutrition Science Institute, Avellino, Italy.

All of the volunteer subjects involved in the in vivo trials gave their informed consent for inclusion before they participated in the study. The study was conducted in accordance with the Declaration of Helsinki, and the protocol was approved by the Ethics Committee of “Federico II” University of Naples—Italy (Project identification code: 101/14; date of approval: 15/09/2014).

### 4.1. Chewing Gum Formulation

Quercetin, flavoring agents, sweeteners, and co-adjuvant excipient amounts were added to the gum-base as reported in Table 2.

The gum base was obtained from Gum Base Company S.p.A. (Lainate, Italy); flavoring agents were supplied from Officina degli Aromi S.r.l. (Milano, Italy); sweeteners were obtained from Sigmar Italia (Almè, Italy). The excipients (magnesium stearate and talc) were obtained from Carlo Erba (Milan, Italy). Qt was supplied from KFO France Co. Ltd., Zhangjiagang, China, and two different concentrations were included in gums: 5 or 10 mg. All other chemicals were of analytical grade and used as commercially obtained.

Gum base, flavoring agents, sweeteners, Qt, and co-adjuvant excipients were mixed in a laboratory mixer (HulaMixer™ Sample Mixer, Waltham, MA, USA) suitable for mixing small batches of powders or dry granules. Sweeteners and flavors were used to obtain a final pleasant taste. Preferably high intensity and not cariogenic sweeteners were selected.

**Figure 6:**
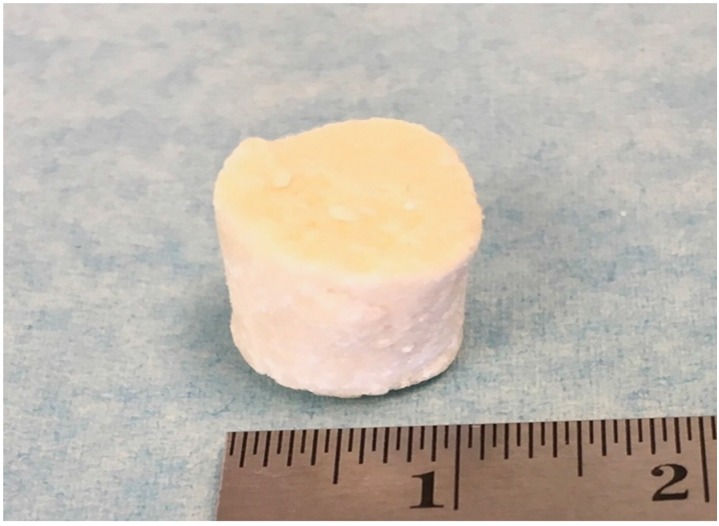
Experimental chewing gums produced, weighing 1.0 g and composed as described in Table 2.

Co-adjuvant excipients (talc, magnesium stearate) were added to the mixture to optimize the compression process. At this point, chewing gums, with their weighed amounts of different mixtures, were produced (Figure 6) by progressively filling the die of a single-punch tableting machine (Matrix 2.2 A, Ataena Srl, Ancona, Italy) and were then compressed at room temperature.

### 4.2. First in Vivo Trial: Release Capability of Enriched Chewing Gums

Both in vivo chewing trials were performed by a group of 36 young volunteers: 18 males and 18 females of mean age 16.3 years. They were recruited among patients followed by the Department of Paediatric Dentistry of Azienda Ospedaliera Universitaria Policlinico “Federico II”, Naples—Italy. The inclusion criteria were good general health (ASA I: Healthy person; ASA II: Mild systemic disease) and an agreement to comply with study procedures. Subjects who had used antibiotics or mouth rinses during the 14 days prior to the beginning of the study were excluded. Only two subjects of the 36 were excluded for this reason. However, during the research period, two subjects chose to end their participation; thus, 32 subjects completed the entire protocol. Participation in the study was voluntary.

The parents of participants also signed an informed consent to participate in the study. The participants were asked not to eat, drink, or chew gums for at least 1 h before the test.

The experimental procedure of the first in vivo trial was so characterized: Volunteers swallowed unstimulated saliva samples before the trial, collected at 5, 10, 20, and 30 min, in order to analyze the saliva flow rate and pH value. Then, they chewed 1.0 g of gum for 30 min at their natural chewing frequency. During this time, the participants collected separate samples, swallowing their saliva after 5, 10, 20, and 30 min into a graduated tube [34,35].

All participants chewed both gums made with the two different Qt contents. The volumes of the saliva samples were recorded, and the flow rates were calculated for each period of chewing; then, the pH of samples was measured with a glass electrode pH-meter at the end of each period [35,36]. The saliva samples were then stored at +4 °C for the chemical assessment of quercetin.

### 4.3. Determination of Quercetin Content in Saliva

The assessment of Qt content in saliva was performed according to the colorimetric method reported by Woisky and Salatino and modified by Chang et al. [37,38]. The amount of Qt was determined after lyophilization, and the residues were re-suspended in methanol/water (80:30, *v*/*v*) solution. Qt pure standard was used to make the calibration curve. Ten milligrams of quercetin was dissolved in 80% methanol and then diluted to 25, 50, and 100 μg/mL. The diluted standard solutions (0.5 mL) were separately mixed with 1.5 mL of methanol, 0.1 mL of 10% aluminum chloride, 0.1 mL of 1 M potassium acetate, and 2.8 mL of distilled water. After incubation at room temperature for 30 min, the absorbance of the reaction mixture was measured at 415 nm with a spectrophotometer UV-DU 730 (Beckman Coulter, Brea, CA, USA). The amount of 10% aluminum chloride was substituted by the same amount of distilled water in the blank. Similarly, 0.5 mL of methanol extracts of samples were reacted with aluminum chloride for determination of quercetin content, as described above.

### 4.4. Second in Vivo Trial: Antimicrobial Activity of Enriched Chewing Gums

A second clinical study was conducted. Starting the experimental period, the collection of saliva samples for each of the 32 subjects was performed (T0) to calculate the density (CFU/mL) of *S. mutans* at baseline. Subsequently, in order to obtain two homogeneous groups, volunteers were divided into two groups of 16 subjects (Groups A and B), having the same concentration of (CFU/mL) of *S. mutans* at baseline.

Two different chewing gums, both 1.0 g in weight, were prepared:

(1) Experimental (Group A): An experimental chewing gum containing 10 mg of quercetin was used. This concentration of Qt was preferred for its highest release in oral saliva during the first minutes of chewing in the first in vivo trial.
(2) Placebo (Group B): The placebo chewing gums were prepared as described above, but without adding quercetin.

Experimental design: All of the Group A participants were asked, after performing oral hygiene, to chew 1.0 g of experimental gum containing 10 mg of Qt for 30 min at their natural chewing frequency, three times a day (after breakfast, after lunch, and before sleeping) for 14 days; the participants of Group B were instructed to chew, after performing oral hygiene, 1.0 g of placebo chewing gum for 30 min, three times a day for 14 days. On the 7th (T1) and 14th (T2) days of treatment, additional salivary samples were collected and immediately incubated to calculate the density (CFU/mL) of *S. mutans* for each subject.

Salivary counts of *S. mutans* in samples were estimated using a chair-side test, and blue mitis-salivarius-agar with bacitracin was used to detect the presence of Streptococci. Saliva was inoculated on a dip-slide with selective media for streptococci. After adding a NaHCO_3_ tablet to the tube, the dip-slides were immediately cultivated at 37 °C for 48 h. The tablet releases CO_2_ on contact with moisture, creating favorable conditions for bacterial growth.

The colonies were identified using a stereomicroscope with 10× magnification, and the culture density (CFU/mL) was visually compared with the aid of a chart provided by the manufacturer. Bacterial colonies were categorized as low (<10^5^ CFU/mL of saliva) or high (≥10^5^ CFU/mL).

### 4.5. Statistical Analysis

At the end of the treatments, the data were processed using the Statistical Package for Social Sciences (version 10.0, SPSS Inc., Chicago, IL, USA). One Way Repeated Measures Analysis of Variance (ANOVA) was used to estimate significant differences in flow rate and pH among the samples collected during use of the chewing gums enriched with 5 and 10 mg of quercetin, in comparison with those of saliva collected during the session without gum chewing. A *p*-value < 0.05 was considered significant.

A regression binary logistic analysis was performed for the second in vivo trial. The statistical significance level was established at *p* < 0.05.

## 5. Conclusions

This study demonstrated that quercetin was released well during the chewing of these new experimental gums, also showing that the gum composition was correctly designed and produced. Moreover, quercetin was effectively released in the oral cavity during the in vivo chewing time, showing an effective antimicrobial concentration in the volunteer’s saliva. Therefore, it is possible to conclude that quercetin included in innovative experimental chewing gums could be efficiently released in vivo during the mastication process in order to combat carious disease. Moreover, it could be interesting to develop new medical devices in order to obtain an extended modulated release of this bioactive molecule in the oral cavity.

## Figures and Tables

**Table 1:** Percentage of samples containing a high density of *Streptococcus* *mutans* CFU (≥10^5^ CFU/mL).

Time	Percentage of Samples Containing High *S. mutans* Density	
	Group A (Test)	Group B (Control)
T0	80.6%	80.6%
T1	50.1%	86.1%
T2	36.1%	77.8%

**Table 2:** Composition (%) of 1.0 g of chewing gum.

Ingredients	Qt0.5 (%)	Qt1.0 (%)
Gum-base	78.5	78.0
Quercetin	0.5	1.0
Flavoring agents and sweeteners	20.0	20.0
Co-adjuvant excipients	1.0	1.0
